# Dissociation and integration of outcome and state uncertainty signals in cognitive control

**DOI:** 10.3758/s13415-023-01091-7

**Published:** 2023-04-14

**Authors:** William H. Alexander, James Deraeve, Eliana Vassena

**Affiliations:** 1 Center for Complex Systems & Brain Sciences, Florida Atlantic University, Boca Raton, FL USA; 2 Department of Psychology, Florida Atlantic University, Boca Raton, FL USA; 3 The Brain Institute, Florida Atlantic University, Boca Raton, FL USA; 4 Department of Experimental Psychology, Ghent University, Ghent, Belgium; 5 Experimental Psychopathology and Treatment, Behavioural Science Institute, Radboud University, Nijmegen, Netherlands; 6 Donders Institute for Brain, Cognition and Behaviour, Radboudumc, Nijmegen, Netherlands

**Keywords:** Anterior cingulate, Anterior insula, Dorsolateral prefrontal cortex, Modeling, Risk

## Abstract

Signals related to uncertainty are frequently observed in regions of the cognitive control network, including anterior cingulate/medial prefrontal cortex (ACC/mPFC), dorsolateral prefrontal cortex (dlPFC), and anterior insular cortex. Uncertainty generally refers to conditions in which decision variables may assume multiple possible values and can arise at multiple points in the perception-action cycle, including sensory input, inferred states of the environment, and the consequences of actions. These sources of uncertainty are frequently correlated: noisy input can lead to unreliable estimates of the state of the environment, with consequential influences on action selection. Given this correlation amongst various sources of uncertainty, dissociating the neural structures underlying their estimation presents an ongoing issue: a region associated with uncertainty related to outcomes may estimate outcome uncertainty itself, or it may reflect a cascade effect of state uncertainty on outcome estimates. In this study, we derive signals of state and outcome uncertainty from mathematical models of risk and observe regions in the cognitive control network whose activity is best explained by signals related to state uncertainty (anterior insula), outcome uncertainty (dlPFC), as well as regions that appear to integrate the two (ACC/mPFC).

Studies investigating brain function during cognitive control frequently observe activity in a constellation of regions in the cognitive control network (Cole and Schneider, 2007), including anterior cingulate cortex and surrounding medial prefrontal cortex (ACC/mPFC), dorsolateral prefrontal cortex (dlPFC), and anterior insular cortex. Activity in these regions is highly correlated (Dosenbach et al., 2006; Hutchison et al., 2012; Seeley et al., 2007) and often follows salient sensory and behavioral events, especially those related to processing behavioral error and task-related feedback. The cognitive control network is densely interconnected, with each region sending and receiving projections from other regions in the network (Augustine, 1996; Barbas and Pandya, 1989; Vogt and Pandya, 1987). Given the high degree of connectivity, and the attendant correlation of activation during behavior, it remains an open question how each of these regions contributes to signaling the need for and deploying control.

The function of all three of these regions has been extensively linked to uncertainty in various forms. ACC activity has been interpreted as indexing response conflict or choice difficulty (Botvinick et al., 2001; Shenhav et al., 2013), tracking the volatility of the environment (Behrens et al., 2007), estimating error likelihood (Brown and Braver, 2005), and predicting the possible outcomes of actions (Alexander and Brown, 2011), all of which correlate with levels of uncertainty. DLPFC function has been associated with learning hierarchical task structure (Badre and D’Esposito, 2009), particularly when learning such structure can reduce uncertainty regarding behavior (Koechlin et al., 2003). Recent theoretical and computational accounts place dlPFC within the framework of predictive coding (Alexander and Brown, 2015, 2018), with dlPFC learning an estimate of the variance around predicted outcomes. Activity in anterior insula has been observed to correlate with outcome risk prediction and risk prediction errors (Preuschoff et al., 2008; Rudorf et al., 2012), and the region has been implicated in categorization (Grinband et al., 2006; Mack et al., 2013) and selective attention tasks when it is necessary to resolve ambiguity in external stimuli (Bach et al., 2009; Deary et al., 2004). These findings point to uncertainty as a primary factor underlying the function of the cognitive control network during behavior.

While it is clear that quantities related to uncertainty are central to brain function, uncertainty is itself an imprecise term (Ellsberg, 1961). One taxonomy of uncertainty organizes uncertainty according to the epistemological status of the underlying probabilities: in risk or expected uncertainty (Huettel et al., 2006; Yu and Dayan, 2005), outcomes are probabilistic, but the probabilities of outcomes are known, or at least knowable, whereas in ambiguity or unexpected uncertainty, outcomes are probabilistic and the probabilities are unknown. Another taxonomy organizes uncertainty according to the variables to which uncertainty applies, regardless of whether the probabilities of a variable having a particular value are known or unknown (Bach and Dolan, 2012). In this classification scheme, multiple decision variables in the perception-action cycle (Fuster, 2001) are subject to uncertainty. Generally, in the framework of machine learning and reinforcement learning (Kaelbling et al., 1998), external stimuli entering the system are used to estimate the current state of the environment, and based on this state estimate, a response can be planned and executed, with consequent outcomes that can be used as the basis of further behavior. *Stimulus uncertainty* can arise from degraded or occluded input to the system, *state uncertainty* can result from ambiguity in the estimate of the current status of the environment, and *outcome uncertainty* reflects the inherent probabilistic nature of a stochastic world. These types of uncertainty are frequently correlated due to cascade effects (Fig. 1): outcome uncertainty may reflect uncertainty in the underlying contingencies of the environment; however, it may be due to uncertainty regarding the current state of the environment; if the context in which a decision maker is operating is not completely known, the outcomes that may be observed also are less certain. Similarly, state uncertainty may be the product of perfect sensory information that nonetheless indicates multiple possible environmental states, or it may be the downstream consequent of noisy input that renders estimates of the state of the environment unreliable. Cascade effects of this sort represent potential confounds on efforts to identify regions of the brain that underlie processes meant to indicate and compensate for various sources of uncertainty in behavior. Although recent work has attempted to identify how state uncertainty influences downstream variables (e.g., dopamine reward prediction errors (Babayan et al., 2018; Mikhael et al., 2022; Starkweather et al., 2018), less is known about how and where state uncertainty may be represented.

**Fig. 1:**
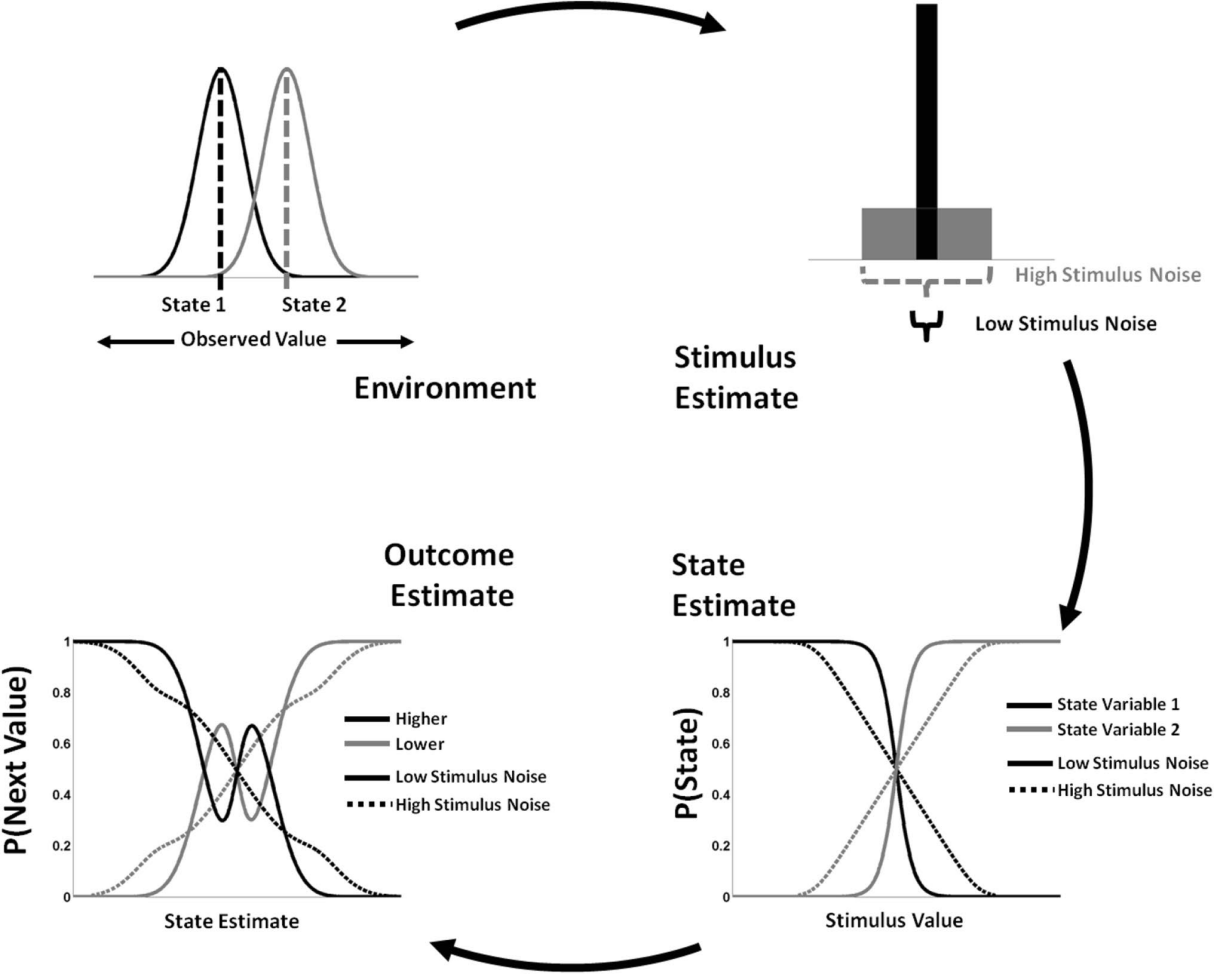
Cascades of uncertainty. Uncertainty may be introduced at multiple points in the perception-action cycle. Given an environment that may be in one of two possible states (upper left frame) and an experimental task to judge whether subsequent values will be lower or higher than an observed value, uncertainty introduced early in the form of stimulus noise (upper right frame) reduces precision in subsequent estimates of the current state of the environment (lower right frame), which in turn leads to increased uncertainty regarding future outcomes (lower left frame)

In this study, we attempt to dissociate neural signatures in the cognitive control network specifically related to state and outcome uncertainty. Of the types of uncertainty described above, state uncertainty remains one of the least well-studied (Bach and Dolan, 2012), possibly due to its confound with both stimulus-related and outcome-related uncertainty. To address these possible confounds, we adapt a multistage, gambling task previously used to investigate the neural correlates of outcome uncertainty (Preuschoff et al., 2008; Rudorf et al., 2012). For each trial, subjects are shown cards from one of two possible decks, which constitute an unobserved state of the environment that may be inferred based on the identity of cards revealed during the trial. Because no effort is made to degrade or mask the cards, we assume that stimulus uncertainty is minimal. To dissociate state and outcome uncertainty, our experimental design uses a previously described mathematical model of risk (Preuschoff et al., 2008). Hence, we are able to identify signals in the cognitive control network related specifically to changes in the level of one source of uncertainty while controlling for the other source.

## Methods

### Subjects

Twenty-two healthy, right-handed volunteers participated in this experiment (8 males). Experimental procedures were approved the UZ Gent Ethics Committee, and all participants gave informed consent and completed safety checklists before entering the scanner to exclude contraindications for participation. Inclusion criteria consisted of normal or correctable-to-normal color vision, and no current use of prescription psychoactive medication. Mean participant age was 23 years (min 20, max 27, standard deviation = 2).

### Behavioral task

**Fig. 2:**
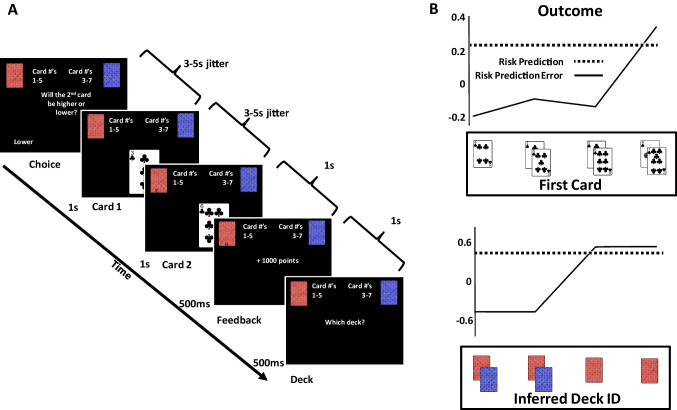
**A)** Experimental task. Subjects were asked to guess whether the second of two successively-presented cards would be higher or lower than the first. Card numbers ranged from 1 to 7 and could be drawn from one of two possible decks on each trial. Cards drawn from the Red deck were numbered 1 to 5, and cards drawn from the Blue deck were numbered 3 to 7. Based on the cards observed during the trial, subjects could infer which deck was used on each trial, and they were asked to identify the deck at the end of each trial. **B)** Outcome and State Uncertainty Signals. Our mathematical model of state and outcome risk, derived from Preuschoff et al. (2008), suggests how Risk Prediction Error (Risk PE) signals following the presentation of the first card might be dissociated for Outcome and State Risk. Outcome Risk PEs (top frame) are equivalent following the presentation of cards 2, 3, 5, and 6; however, for cards 3 & 5, the identity of the deck cannot be inferred, whereas for cards 2 and 6, deck identity can be inferred. For equal Outcome Risk PEs, the risk model predicts differing levels of State Risk PE (bottom frame)

Subjects performed a gambling task adapted from Preuschoff et al. (2008) (Fig. 2A). For each trial of the task, subjects were shown two cards in sequence, separated by a delay. Before observing the first card, subjects were asked to guess whether the second card would be higher or lower than the first. Because subjects had no information on which to base their guess, the probability of guessing correctly on a trial was 0.5.

Subjects were informed that there were two decks (red and blue) used in the experiment. Each deck contained five cards: the red deck contained cards ace through 5, and the blue deck cards 3 through 7. Subjects were additionally informed that on each trial, only one of the two decks would be used, that both cards shown during the trial would be drawn from that deck, and that, based on the cards shown, they may be able to determine the identity of the deck from which they were shown cards on a given trial. The experiment was arranged such that on one third of the trials, card number 4 would be the first card displayed (with a 50% chance the card was drawn from either the red or the blue deck). For the remaining trials, each card had an equal chance of being displayed first. Because there was an equal chance of observing each card (besides card number 4), effects of novelty or salience were excluded as possible causes of differences in activity.

After indicating whether they thought the second card would be higher or lower than the first, subjects were shown the first card, followed by the second after a randomly jittered interval (3,000-5,000 ms). Subjects then received feedback indicating whether their guess was correct or incorrect, as well as the number of points won (+1,000) or lost (−1,000), respectively. After subjects received feedback, they were then asked to identify which deck they had received cards from during the trial. If subjects correctly identified the deck, the number of points they lost (if they guessed incorrectly) was reduced by 500. If subjects incorrectly identified the deck, the number of points subjects won (if they had guessed correctly) was decreased by 500. Following feedback indicating the final number of points won or lost, the next trial began after a jittered interval (3,000-5,000 ms).

### fMRI data acquisition

While performing the behavioral task described above, subjects underwent functional MRI scanning in a Siemens 3T Magnetom Trio MRI Scanner with a 32-channel, radiofrequency head coil. Both structural (T1-weighted MPRAGE sequence, 176 high-resolution slices, TR = 1550ms, TE = 2.39, voxel size = 0.9 mm X 0.9 mm X 0.9 mm, FOV = 220 mm, flip angle = 9°), and functional (T2-weighted EPI sequence, 33 slices, TR = 2,000 ms, TE = 30 ms, voxel size = 3 mm X 3 mm X 3 mm, FOV = 192 mm, flip angle = 80°). Approximately 1,400 volumes per subject were collected over 50 minutes while subjects performed the task.

### fMRI data preprocessing

Data were analyzed using SPM12 (http://www.fil.ion.ucl.ac.uk/spm). Scanning sessions were divided into 4 runs, and the first 4 volumes of each functional run were discarded to allow for a steady-state magnetization. Functional images were aligned to the first image of the run, and the T1-weighted image was co-registered to the functional mean image for normalization, performed through SPM12’s unified segmentation and nonlinear warping approach. Images were aligned to the MNI template (Montreal Neurological Institute) and were smoothed by using a Gaussian kernel (8-mm FWHM).

### fMRI analysis

A total of five GLMs (GLMs 1-5; Table 1) were created to conduct model-based analysis of fMRI data. The first two GLMs looked specifically at effects of Outcome and State risk prediction errors (Risk PE) alone. Each of these GLMs contained 108 regressors (27 regressors for each run); the regressors for each run included 24 movement regressors (Power et al., 2014) (pitch, yaw, roll, and X, Y, & Z translation, scan-to-scan differences, and the squared values of each), and 1 block-wise regressor. Each trial, aligned to the presentation of the first card in the sequence, was modeled as a single regressor (duration = 0 ms), which was parametrically modulated by values obtained from a mathematical model of risk for outcome and state uncertainty (see below). The delay between presentation of the first and second cards was jittered, and we did not expect effects from the second card presentation to have an influence on effects related to the presentation of the first card; for this reason, the second card presentation was not modeled in our GLMs. For the first GLM (GLM1), values for the parametric modulator were Risk PEs from a risk model in which the deck identity (red/blue) of the trial was to be predicted. For the second GLM (GLM2) while the values of the parametric modulator were Risk PEs from the risk model in which the outcome (win/lose) was predicted.

**Table 1:** General linear models

	Order of parametric modulators		
	Modulator 1	Modulator 2	Modulator 3
GLM1	State Risk PE	N/A	N/A
GLM2	Outcome Risk PE	N/A	N/A
GLM3	State Risk PE	Outcome Risk PE	N/A
GLM4	State Risk PE	Outcome Risk PE	Reward PE
GLM5	Outcome Risk PE	Reward PE	N/A

## Parametric modulators modulate an event Regressor modeling the onset of the first card in each trial

The purpose of including a single parametric modulator in GLM1 and in GLM2 was to identify which regions, if any, significantly correlated with both forms of Risk PE, allowing comparison of the contributions of deck identity and outcome Risk PE modulators. The values for both types of Risk PE are correlated, being minimal when the first card delivers no information about deck or outcome identity (i.e., when card 4 is presented), and maximal when presentation of the first card indicates the outcome and deck identity with perfect certainty (i.e., cards 1 and 7). Given this correlation it is possible that, when considered alone, each form of Risk could correlate with activity in similar regions of the brain, with attendant implications for studies in which only outcome or state risk alone are considered.

In GLM 3, both State and Risk PEs are included as parametric modulators. By default, SPM 12 serially orthogonalizes modulators, leading to potential issues with interpretation (Erdeniz et al., 2013; Mumford et al., 2015). To avoid this, serial orthogonalization was disabled in our analyses—each parametric modulator “competed” with the others to explain variance. Where GLMs 1 and 2 effectively assigned all variance possible to either the State or Outcome Risk PE modulators, in GLM 3 the variance assigned to each modulator is only that which can be uniquely accounted for by that modulator.

To investigate possible effects of *reward* (as opposed to risk) prediction errors on activity in the regions identified by GLM 1-3, two additional GLMs were created which included reward prediction error (eq. 2 below) as a parametric modulator. GLM4 was identical to GLM3 above, except that it included reward prediction error as a third parametric modulator, for a total of 116 regressors. GLM 5 directly tested whether activity in any brain region was related specifically to signed reward predictions errors versus outcome Risk PEs, and was identical to GLM1 above, except that the State Risk PE modulator was replaced by a reward prediction error modulator.

### Mathematical models of risk

Preuschoff et al. (2008) introduced a mathematical model of risk, based on reward prediction, that we adapt to compute changes in state uncertainty. The Preuschoff model begins with an estimate of the probability of winning or losing on each trial. At the beginning of each trial, subjects make guesses without any information, leading to an equal likelihood of guessing correctly or incorrectly. In our experiment, the final outcome of a trial depends both on whether the subject correctly guessed if the second card would be higher or lower than the first but also on the subject’s ability to correctly identify the deck from which cards were drawn: correctly identifying a deck on a losing trial reduces the number of points lost from −1,000 to −500, while incorrectly identifying a deck on a winning trial reduces the number of points won (+1,000 to +500).

1 $${EV}_0=\left({P}_{win}\times {Value}_{win}-{P}_{incorrect}\times {Value}_{incorrect}\right)+\left({P}_{lose}\times {Value}_{lose}-{P}_{correct}\times {Value}_{correct}\right)$$

We assume that when subjects observe a card that unambiguously indicates one deck or another (i.e., cards 1, 2, 6, and 7) that they will always correctly identify the deck. Conversely, when the cards presented do not indicate one deck or the other, we assume that subjects guess each deck equiprobably.

Following the presentation of the first card, expected value may be updated depending on new information: observing the ace or the seven, for example, eliminates all uncertainty, and the expected value is now either the value of winning or losing, depending on the subject’s guess. The change in expected value produces a *reward prediction error:*

2 $${PE}_{rew}={EV}_1-{EV}_0$$

Risk prediction values build from reward predictions and reward prediction errors. While the expected value of a trial, before observing any cards, is always equal to 0 in this experiment, observation of the first card produces a number of different new expected values, along with attendant reward prediction errors. Risk prediction in the model is defined as the expected squared reward prediction error. Hence, before any cards are observed, the risk prediction is

3 $${ER}_0=\sum\nolimits_{n=1}^7P(n)\times {\left[\left({EV}_1|n\right)-{EV}_0\right]}^2$$

*Risk prediction errors* are calculated as the difference between the squared reward prediction error (Eq. 2) and the expected squared reward prediction error (Preuschoff et al., 2008)

4 $${PE}_{risk}={PE}_{rew}^2-{ER}_0$$

The above equations formalize a mathematical model of risk as it relates to *outcomes.* Reward and risk prediction signals are defined in terms of the expected value (number of points) and changes in the expected value as it evolves over the course of a trial. The above equations can be adapted to the case of state (as opposed to outcome) prediction by substituting the predicted identity of the deck being used in place of value:

5 $$Expected\ State={P}_{red}\times 1+{P}_{blue}\times -1$$

where 1 and −1 code for deck identity. At the beginning of each trial, each deck is equally likely, and the expected state has a value of 0. Following the observation of the first card, a state prediction error is calculated as in Eq. 2, as well as state risk and state risk prediction error (Eqs. 3 and 4).

When applied to the contingencies of our experiment, the mathematical models of outcome and state risk yield predictions regarding the pattern of neural signals associated with State and Outcome Risk PE following presentation of the first card (Fig. 2B). Specifically, for both state and outcome risk, Risk PEs are expected to be lowest following the presentation of card number 4, which carries no information regarding deck identity, nor does it change the probability of a guess being correct or incorrect. Similarly, the model predicts that activity will be highest following presentations of cards 1 and 7, both of which are diagnostic with respect to deck identity as well as the correctness of the subjects guess. Critically, prediction errors for outcome risk and state risk diverge following presentation of cards 2/6 and cards 3/5.

## Results

### Behavioral results

In our task, subjects were required to make two behavioral responses: 1) they were asked to guess whether the second of two cards would be higher or lower than the first, and 2) they were asked to identify the deck (red or blue) from which they received cards on each trial. Overall, subjects performed as expected. Although specific correct/incorrect outcomes rates were not enforced, average accuracy for high/low guesses was at chance over all subjects (P(correct) = 0.49), and subjects were able to identify the deck with high accuracy on those trials in which the deck ID was identifiable (P(correctID) = 0.99). The median number of points earned for the entire session was 45,500. Analysis of individual performance suggested that, while as a group, subjects performed as expected, some individuals performed substantially below chance levels. A binomial test on high/low choice accuracy revealed three subjects whose performance was significantly lower than chance (*p* < 0.025). These subjects were excluded from our fMRI analyses. A fourth subject ended the scanning session early, and the incomplete data collected from this subject was also not included in our analyses. A total of 18 subjects were therefore used in our fMRI analyses.

### Model-based results

Our analysis of fMRI data attempted to answer three questions of increasing specificity. Our first goal was to identify regions of the brain that correlated with Risk PEs irrespective of whether this correlation related specifically to Outcome or State Risk PE. Second, we sought to test whether State or Outcome Risk PEs explained brain activity in any region after accounting for effects unique to the other Risk PE, as well as the variance explained by both. Finally, we tested whether any region showed effects *specific* to either State or Outcome Risk PEs. Whereas our second question asks whether effects unique to State or Outcome Risk PEs explain additional variance in the BOLD signal, our third questions asks whether State (Outcome) Risk PEs explains significantly more variance than Outcome (State) Risk PEs. Another way to state this is that our second question examines whether State/Outcome Risk PE effects are different from 0, whereas our third question examines whether State and Outcome Risk PE effects are different from each other. Because our experimental design is derived explicitly from previous studies observing Outcome Risk PE-related activity in bilateral anterior insula (Preuschoff et al., 2008; Rudorf et al., 2012), we have a strong *a priori* hypothesis that Risk PE-related activity will be observed in those areas.

Risk PE values from the models of Outcome and State risk were regressed against BOLD activity time-locked to the onset of the first card displayed in each trial. In the Outcome model, reward and risk prediction errors derive from updates in the expected number of points to be gained or lost at the conclusion of the trial. Conversely, in the State model, predictions and prediction errors are due to changes in the ability of the subject to divine the current state, here conceived as the identity of the deck from which the subject is shown cards on each trial. Hence, the two models suggest different but partially correlated patterns of activity that may be observed following the presentation of the first card (Fig. 1B).

**Fig. 3:**
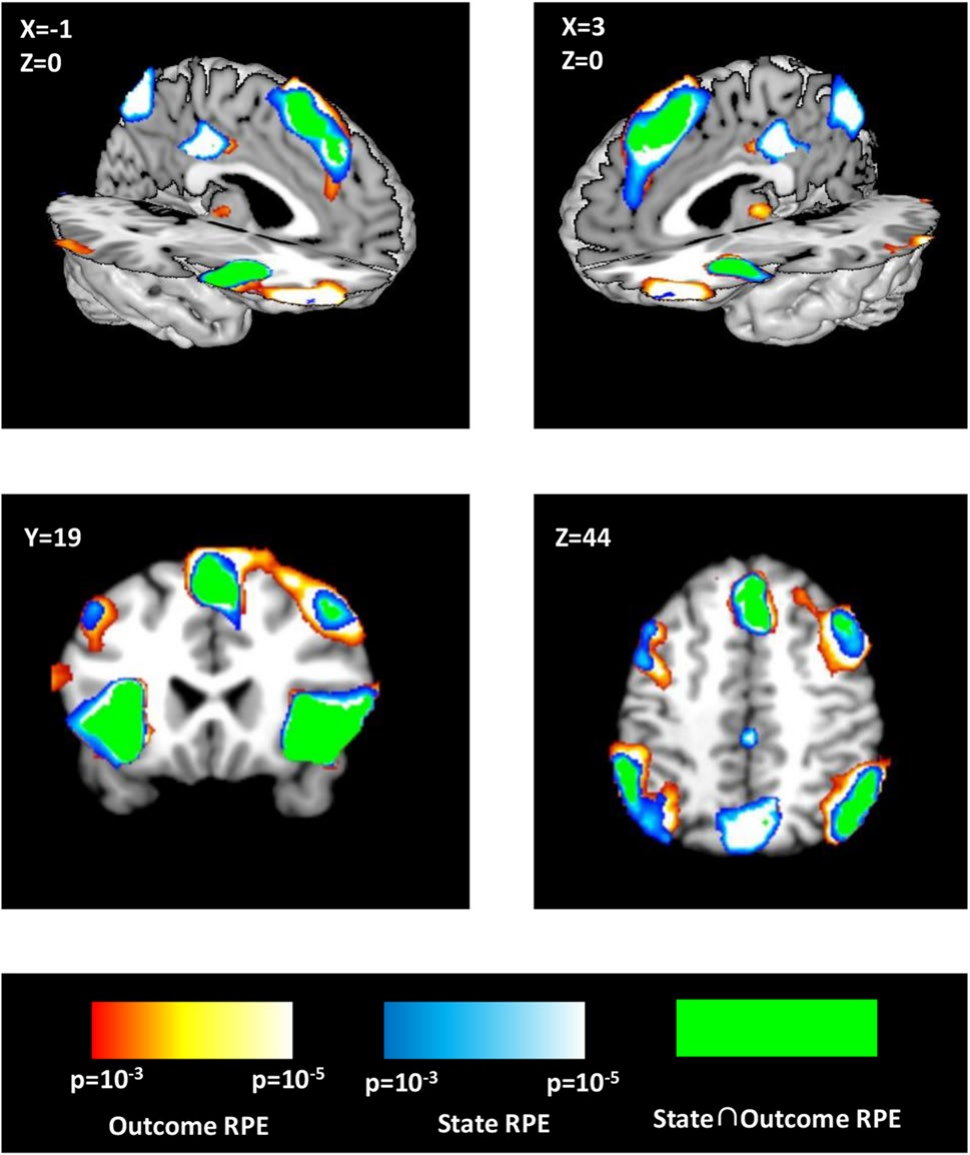
Risk Prediction Error in the Cognitive Control Network. Values derived from the mathematical model of risk for State and Outcome Risk PEs correlate with activity in regions commonly associated with cognitive control and decision-making. Blue indicates voxels correlating with State Risk PE (GLM 1), red with Outcome Risk PE (GLM 2), and green the overlap for voxels surviving an uncorrected threshold of *p* < 0.001 for both State and Outcome Risk PE. Regions observed to correlate with both types of Risk PE include ACC/mPFC, anterior insula, parietal, and right caudal dlPFC (BA 9). Outcome Risk PE selectively correlated with bilateral rostral dlPFC, as well as left BA 9, although this latter result did not survive FWE (*p* < 0.05) correction at the cluster level

With GLMs 1 and 2 (Table 1),we first investigated regions of the brain whose activity correlated with Outcome and State Risk PEs without regard for whether these effects are specific to one or the other. These results (Table 2) reveal a set of regions associated with the cognitive control network (Fig. 3), showing a positive linear relationship with Outcome and State Risk PE modulators. These regions include bilateral anterior insula and ACC/mPFC. Additionally, regions in bilateral rostral dlPFC (BA 46/47) and right caudal dlPFC (BA9) passed whole-brain cluster-level correction (voxel threshold *p* < 0.001, cluster-level FWE threshold < 0.05) only for the Outcome Risk PE modulator from GLM2, providing an early indication that these regions may code specifically for Outcome Risk PEs.

**Table 2:** Individual modulators

Region	Peak coords			Peak Voxel t-stat (df = 17)	Cluster statistics		
	X	Y	Z		P(FWE)	P(FDR)	Extent
State Risk PE (GLM1)							
Right anterior insula	34	18	-6	8.63*	<0.001	<0.001	863
ACC/mPFC	4	22	48	6.54*	<0.001	<0.001	659
Left anterior insula	-32	20	10	7.40*	<0.001	<0.001	517
Posterior cingulate	0	-28	34	6.19*	0.001	<0.001	251
Right parietal	44	-68	42	5.02	<0.001	<0.001	288
Left parietal	-56	-44	46	4.94	0.002	0.001	216
Precuneus	10	-56	38	5.25	<0.001	<0.001	578
Outcome Risk PE (GLM2)							
Right anterior insula	36	18	-6	8.45*	<0.001	<0.001	593
Left anterior insula	-30	20	10	6.86*	<0.001	<0.001	390
Right caudal DLPFC	42	10	52	7.51*	<0.001	<0.001	480
Right rostral LPFC	44	52	0	7.44*	<0.001	<0.001	449
Left rostral LPFC	-44	50	-6	5.55	<0.001	<0.001	239
ACC/mPFC	4	24	50	6.41*	<0.001	<0.001	647
Right parietal	54	-50	46	6.83*	<0.001	<0.001	794
Left parietal	-56	-44	46	6.06*	<0.001	<0.001	367
Left mid DLPFC	-50	40	26	5.43	0.0004	0.005	96

*p(FWE) < 0.05, whole-brain correction

**Fig. 4:**
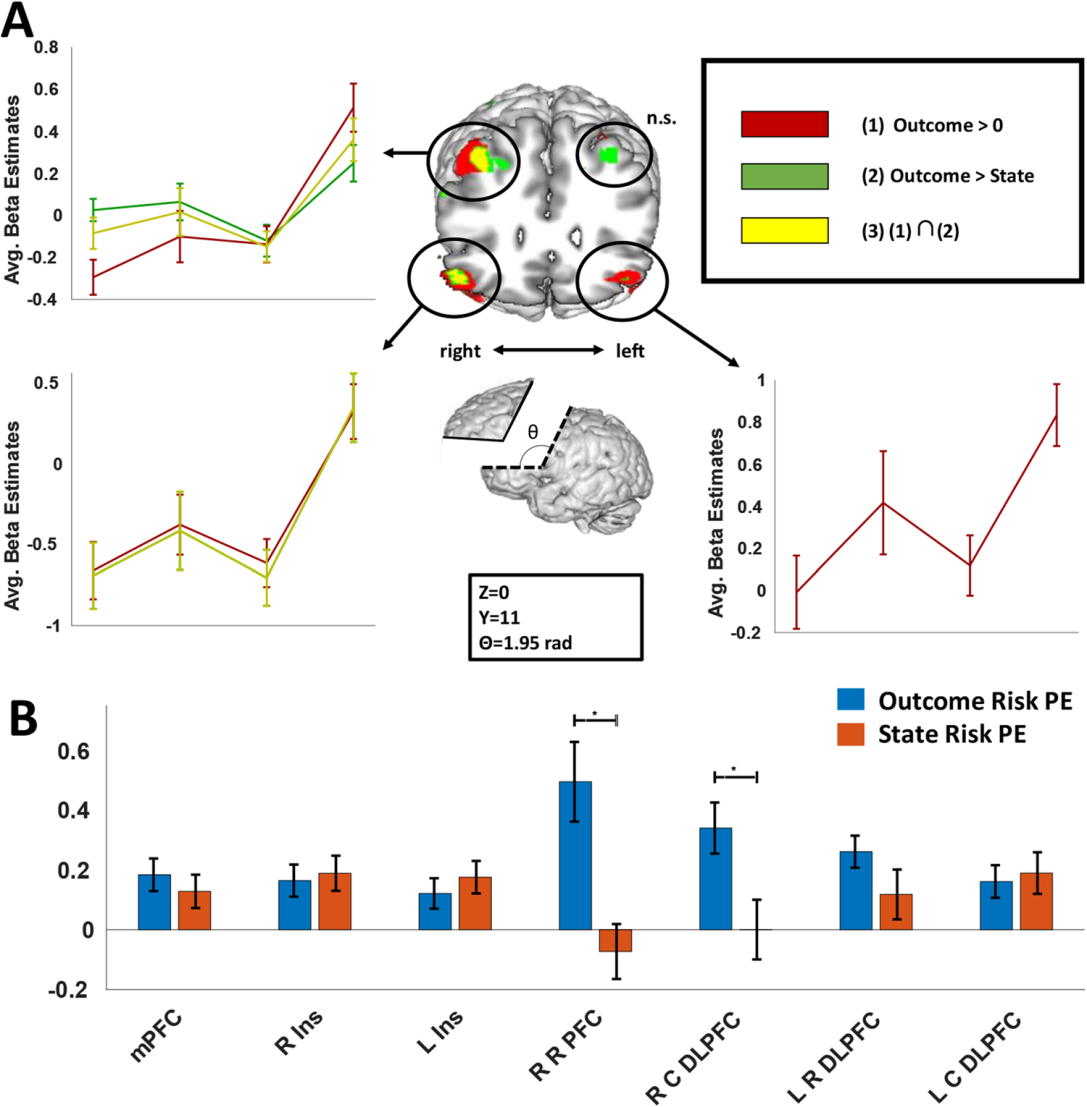
Outcome Risk PE-specific activity. **A)** Activity in regions in bilateral rostral lPFC and caudal dlPFC corresponded to Outcome Risk PE signals derived from the mathematical model of risk. The number of voxels surviving threshold (uncorrected ***p*** < 0.001) was greater for comparisons of Outcome Risk PE versus State Risk PE than for comparison of Outcome Risk PE versus 0, indicating the State Risk PEs were slightly anti-correlated with activity in these regions. This is especially apparent in left caudal dlPFC, in which activity is more in line with a negative State Risk PE signal than for Outcome Risk PEs. **B)** Significant interaction between Risk Type and Region appears to be driven by increased activity associated with Outcome Risk PEs in right lateral PFC

We then investigated whether activity in any of these regions could be explained by State or Outcome Risk PEs after accounting for shared effects by including both values in the same GLM (GLM 3). By default, SPM12 orthogonalizes successive parametric modulators with respect to previous modulators, potentially introducing problems with interpretation. To avoid this, we disabled serial orthogonalization in our analyses; effects observed were related only to the variance each parametric modulator could uniquely account for. We observed a subset of the clusters from the previous analysis that positively relate to the Outcome Risk PE modulator (Fig. 4A; Table 3; bilateral BA46/47, right BA 9). Conversely, we observed significant clusters in bilateral anterior insula that were positively related to State Risk PE. Notably, only Outcome Risk PE effects were observed ACC/mPFC.

**Table 3:** GLM3

Region	Peak coords			Peak Voxel t-stat (df = 17)	Cluster statistics		
	X	Y	Z		P(FWE)	P(FDR)	Extent
State Risk PE							
Right anterior Insula	34	18	4	5.09	0.001	0.002	215
Precuneus	2	-78	40	4.99	0.033	0.021	106
Left anterior insula	-34	18	10	5.30	0.045	0.021	97
Outcome Risk P							
Left rostral LPFC	-38	48	2	4.64	0.003	0.002	193
Right rostral LPFC	44	50	0	5.74*	<0.001	<0.001	641
Right caudal DLPFC	40	12	48	6.27*	<0.001	<0.001	430
Right parietal	50	-66	38	5.54	<0.001	<0.001	669
Dorsal mPFC	8	26	52	4.94	<0.001	<0.001	261
Right anterior insula	34	20	-4	4.54	0.012	0.008	138

Finally, we tested whether State or Outcome Risk PEs were specific (State Risk PE > Outcome Risk PE or Outcome Risk PE > State Risk PE) to any region by performing a paired t-test on the average (across runs) beta parameters estimated for each subject for the State and Outcome Risk PE modulators (Table 4). At the whole-brain level, only visual cortex was significant for a unique effect of State Risk PE over Outcome Risk PE. We observed activity correlating with Outcome Risk PEs in regions in the right hemisphere, including BA 46/47, right BA 9, and parietal cortex, significant after cluster correction (p(FWE) < 0.05, voxel threshold *p* < 0.001), suggesting that these regions uniquely encode uncertainty related to outcomes.

**Table 4:** Comparison of modulators

Region	Peak coords			Peak Voxel t-stat (df = 17)	Cluster statistics		
	X	Y	Z		P(FWE)	P(FDR)	Extent
State Risk PE > Outcome Risk PE							
Visual cortex	4	-84	14	7.07	<0.001	<0.001	1759
Outcome Risk PE > State Risk PE							
Right parietal	16	-26	50	5.95	<0.001	<0.001	526
Right caudal DLPFC	34	8	46	5.72	<0.001	0.001	318
Right rostral DLPFC	44	48	-2	4.76	0.004	0.011	179

To test for an interaction of Risk PE type and region, we defined six ROIs based on the overlap between regions in the cognitive control network with activity correlating with either Outcome or State Risk PEs (GLMs 1 and 2). We entered the beta values for each subject averaged across each ROI for State and Outcome Risk PEs from GLM3 in a two-way ANOVA (Risk Type x ROI). Results showed a main effect of condition (Outcome > State; F(1,216) = 15.91, *p* = 0.0001), as well as a significant interaction between region and risk type (F(5,216) = 4.78, *p* = 0.0004). Pairwise comparisons (Fig. 4B) suggest that this interaction was driven by right DLPFC—activity associated with Outcome Risk PEs was greater that State Risk PEs for both rostral (t(17) = 2.208, *p* = 0.011) and caudal (t(17) = 2.21, *p* = 0.040) DLPFC.

### Anterior insula and outcome uncertainty

**Fig. 5:**
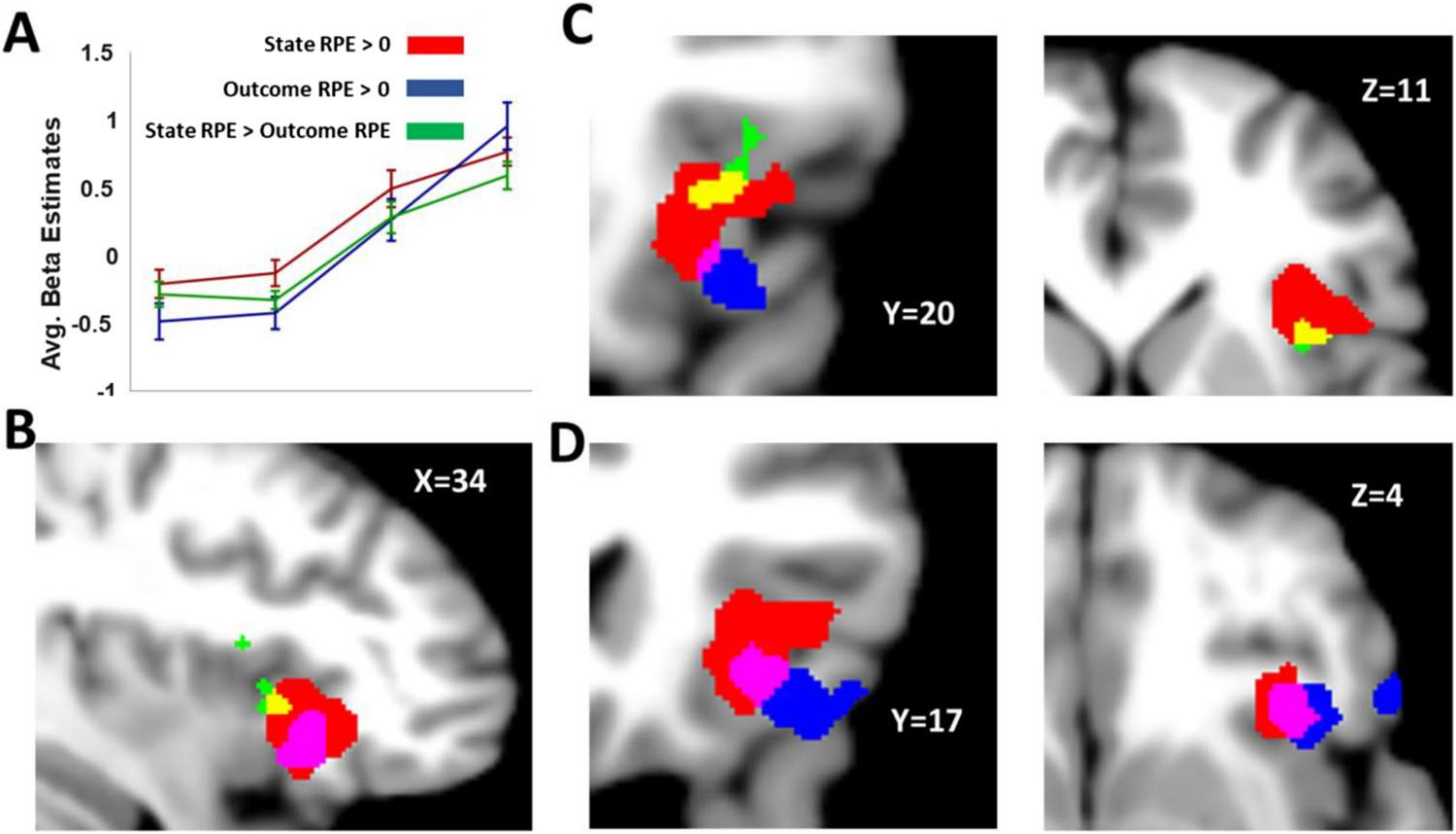
State risk prediction errors in right anterior insula. Signals corresponding uniquely to State Risk PEs (green/yellow) were observed in right dorsal anterior insula cortex (small volume correction p(FWE) < 0.05). Signals related to Outcome Risk PEs (blue) were also observed ventrally to State Risk PEs (voxel threshold < 0.001), consistent with previous observations (Preuschoff et al., 2008; Rudorf et al., 2012); however these signals were not uniquely explained by Outcome Risk PEs, and overlapped regions in anterior insula whose activity was also explained by State Risk PEs (magenta)

Signals corresponding both to State Risk and Outcome Risk PEs passed whole-brain corrections within right anterior insula. However, after applying whole brain corrections, no voxels appeared to *uniquely* encode either type of Risk PE (i.e., for no voxel was the effect of State Risk PE greater than Outcome Risk PE or vice versa). Because the design of our experiment derives explicitly from previous studies that observed Outcome Risk PE signals in anterior insula, we conducted additional analyses exclusively in anterior insula to further assess possible differences in signaling state or outcome risk. We defined an anatomical volume for bilateral anterior insula (wfupickatlas, dilation = 2, bounded at the posterior extent of the central sulcus of the insula (Mutschler et al., 2013)). Within this volume, we tested for differences between State and Outcome Risk PEs by conducting a paired t-test of the averaged beta values for State and Outcome Risk PEs from GLM3. No significant voxels or clusters were observed for the Outcome Risk PE > State Risk PE comparison. For the State Risk PE > Outcome Risk PE comparison, we observed a cluster of voxels in right insula (Fig. 4, green/yellow) whose activity was better explained by the State Risk PEs than Outcome Risk PEs at a significance threshold of *p* < 0.001 (peak voxel MNI Coordiantes 38, 14, 12, t(17) = 5.3, p(FWE) = 0.025, cluster-level p(FWE) = 0.006, voxel extent = 74). These results suggest that, although Outcome Risk PEs can explain activity in subregions of anterior insula beyond that explained by State Risk PEs, no voxels appeared to uniquely code for Outcome Risk PEs Fig. 5.

### State and outcome risk PEs in anterior cingulate

In our first analysis (GLMs 1 and 2), a cluster of voxels in ACC/mPFC correlating with both (uncontrolled) State and Outcome Risk PE) was observed (Fig. 3). A related cluster was observed for Outcome Risk PEs in this region (Table 3) even after State Risk PEs were considered (GLM 3). However, no regions within mPFC exhibited *unique* effects of either State or Outcome Risk PEs, i.e., activity associated with Outcome Risk PEs was not significantly different from activity associated with State Risk PEs. One possibility is that activity in this cluster reflects a reward prediction error, as distinct from errors in risk prediction. In order to test this possibility, we created an additional GLM which included reward prediction error as a parametric modulator in addition to State and Outcome Risk PEs (GLM 4) and another GLM in which only Outcome Risk PEs and reward PEs were included (GLM 5). Analysis within the ACC ROI (cluster-corrected, voxel threshold *p* < 0.001) revealed no significantly active voxels for either positive or negative effects of reward prediction errors, nor were any voxels observed for a more lenient threshold of *p* < 0.01. Together, the results of GLM4 and GLM5 suggest that the cluster observed in ACC/mPFC is not specific to State Risk PE, Outcome Risk PE, nor reward prediction errors.

## Discussion

The results of this study suggest how uncertainty about different task variables is represented and integrated in the cognitive control network. Uncertainty can arise at multiple points in the neural processing stream (Bach and Dolan, 2012): external input to the system may be noisy (stimulus uncertainty), the operating context in which an agent makes decisions may be unknown (state uncertainty), and the outcomes of decisions may be probabilistic rather than deterministic (outcome uncertainty). Uncertainty at early points in the stream can have cascade effects that contribute to uncertainty at later points, resulting in a confound amongst various types of uncertainty (Fig. 1). Given this possibility, controls are needed in order to disentangle the neural regions involved processing uncertainty related to different decision variables. In this study, we minimize stimulus uncertainty—task-relevant stimuli are presented clearly without degradation or masking, while dissociating state and outcome uncertainty. By dissociating state and outcome uncertainty, we sought to identify brain regions predominantly involved in processing one form of uncertainty over the other.

Processing of state uncertainty in this study appears to primarily involve right anterior insula, a region frequently implicated in cognitive tasks, such as categorization (Grinband et al., 2006; Mack et al., 2013), resolving stimulus ambiguity (Bach et al., 2009; Deary et al., 2004; Ho et al., 2009), salience (Menon and Uddin, 2010; Seeley et al., 2007; Wiech et al., 2010), and allocating attention (Downar et al., 2002; Eckert et al., 2009; Nelson et al., 2010). Our findings do not directly contradict these previous results but offer a complementary interpretation. Considering that stimulus uncertainty can have downstream effects on state and outcome uncertainty, the activation of anterior insula in response to noisy or ambiguous stimuli can be viewed as a consequence of uncertainty in state induced by stimulus-related uncertainty. Similarly, our results provide an additional perspective on studies that observed activity in insula correlated to Outcome Risk PEs (Preuschoff et al., 2008; Rudorf et al., 2012). In those studies, the only type of uncertainty investigated related specifically to outcomes (win/lose), while state uncertainty was not explicitly manipulated. In this study, we observe signals related to Outcome Risk PE in anterior insula as well as signals related to State Risk PE. One possibility is that the processing of state and outcome uncertainty is not unique to any specific region (Eisenreich et al., 2017) but that regions involved in processing uncertainty do so for multiple types of uncertainty, albeit weighted toward one type over another. In the absence of a source of state uncertainty in previous studies, then, it may be that signals related to outcome uncertainty are the principal component of activity in anterior insula. A second possibility is that states and outcomes are not cleanly separable concepts. In our experiment, state was conceived as the identity of the deck from which cards were drawn; however, state might also be thought of as an estimate of the condition of the deck before any information being revealed, e.g., whether the environment is in a state such that the second card will be higher than the first or vice versa. Under this notion of state, then, successive card presentations allow for more refined estimates of the initial state of the environment. These estimates may be indistinguishable from estimates of the eventual outcome of the trial.

Outcome Risk PEs, considered on their own, engaged regions in lateral PFC, including bilateral BA 46/47 and right BA 9. A large body of literature has associated these regions, and dlPFC more generally, with working memory and representing hierarchical task structure (Alexander and Brown, 2015; Badre, 2008; Badre and D’Esposito, 2007, 2009; Koechlin et al., 2003). Considering our experiment incorporated two levels of uncertainty related to state and outcome, a reasonable *a priori* hypothesis would be that activity in lPFC may have followed a hierarchical gradient, with changes in more abstract (i.e., state) uncertainty activating more rostral aspects of lPFC, and concrete (outcome) uncertainty changes activating more caudal aspects. In studies investigating the hierarchical organization of prefrontal cortex, it is frequently the case that hierarchically-structured information is successively integrated to govern future behavior: context cues govern how cues related to rules and task-sets are interpreted, and these in turn influence processing of concrete response cues (Collins and Frank, 2013; Koechlin et al., 2003; Nee et al., 2013; Nee and D’Esposito, 2016). Information is therefore, in a sense, updated proactively, with the purpose of contextualizing subsequent cues and behavior. In this study, in contrast, subjects are informed that a single deck is selected for use in each trial, and information gained following the presentation of cards can be used to discern which deck is in use. In this case, information gained following the presentation of each card may not be exclusively used for proactive updates of expectations, but also to update retroactively the estimates of state identity. Our finding that State Risk PE signals correlated only with activity in insula (while Outcome Risk PE signals were observed in lPFC as well as insula) may therefore relate to the distinction of ventral control pathways involved in reactive information integration and dorsal control pathways involved in proactive prediction and behavior (Tops and Boksem, 2012).

When activity associated with Outcome Risk PEs was compared with State Risk PE activity, we observed significant regions exclusively in the right hemisphere of the brain, including lateral PFC and parietal cortex. Similarly, we observed activity uniquely related to State Risk PEs in right anterior insula. Lateralization of attentional processes in the right hemisphere has been reported in humans and monkeys (de Schotten et al., 2011), and may be related to executive processes underlying the control of attention (Spagna et al., 2020). While the present results do not offer new perspectives on *why* control effects may be right-lateralized, they may suggest that representation of control-relevant variables may be more precise in right hemisphere. That is, although regions in both hemispheres coded for State and Outcome Risk PEs to some extent, representations of one or the other were only distinguishable in right hemisphere.

Finally, although ACC/mPFC activity correlated with risk prediction errors, it favored neither state- nor outcome-related signals, nor signals related to reward prediction errors. Instead, activity in the region appeared to broadly integrate Outcome and State Risk PEs. While the BOLD signal recovered from ACC/mPFC appears to be a composite of different types of Risk PE signals, it is not necessarily, nor even likely, the case that the activity of individual neurons reflects the composite signal. First, it is a frequent observation that neurons with drastically different activity profiles exist in an interdigitated fashion in ACC (Sallet et al., 2007), and the co-activation of these neurons during behavioral episodes can give rise to ensemble activation that does not reflect underlying neural computations (Alexander and Brown, 2011, 2014; Botvinick et al., 2001; Stuphorn and Schall, 2006). Second, it is unclear what the functional import of a signal compositing different types of uncertainty would be. Absent any compelling rationale for such a combined signal, these findings are broadly consistent with models of ACC/mPFC that suggest that individual neurons in the region code for discrepancies between expected and observed events (Alexander and Brown, 2011).

Although we did not restrict our analyses to specific brain areas *a priori*, we observed activity correlated with State or Outcome Risk PEs primarily in regions typically associated with the cognitive control network. However, as noted in the *Introduction*, state and outcome uncertainty are only two possible dimensions along which uncertainty-related brain activity might be decomposed. We specifically note stimulus and response uncertainty (Fig. 1A) as additional sources of uncertainty that might be tracked by the brain. A strong possibility is that brain regions implicated in signaling prediction errors, which we do not observe here, may instead respond to prediction errors along these alternate dimensions. However, future work controlling for these additional forms of risk prediction errors will be needed.

While we characterize the brain areas observed in this study as belonging to the “cognitive control” network (Cole and Schneider, 2007), other work has identified these regions as belonging to, variously, the salience network (Seeley et al., 2007), the task-positive network (Fox et al., 2005), the frontoparietal network (Vincent et al., 2008), or the multiple-demand network (Camilleri et al., 2018). In our view, the specific nomenclature used matters less than understanding how the regions that make up these networks respond differentially to various forms of uncertainty. That said, state and outcome uncertainty may nonetheless correlate with alternative interpretations of the regions we observe in this study. For example, activity in ACC/mPFC is frequently interpreted as indexing conflict (Botvinick et al., 2001) or choice difficulty (Shenhav et al., 2013), quantities that are maximal when uncertainty about future outcomes is highest. Similarly, discrimination difficulty has been found to elicit activity in parietal cortex (Hagen et al., 2006). Although there is considerable overlap in the formalization of such alternative interpretations, we believe that uncertainty provides a convenient way of formalizing the activity of the various regions we observe under a common framework.

Activity across the regions we report in this study is frequently correlated, especially following salient, behaviorally relevant events, such as behavioral error (Bastin et al., 2017; Dosenbach et al., 2007; Gläscher et al., 2012; Ham et al., 2013; Hester et al., 2004; Hutchison et al., 2012; Seeley et al., 2007). Due to this ubiquitous co-activity, determining the contribution of each region to cognition and behavior remains a significant challenge for cognitive neuroscience (Cieslik et al., 2013; Dosenbach et al., 2007; Gläscher et al., 2012; MacDonald et al., 2000), particularly with regard to anterior insula and ACC (Craig, 2009; Critchley et al., 2004; Gu et al., 2010). Recent evidence from intracerebral EEG recordings in human suggest a causal role of error signals in anterior insula on activity in ACC/mPFC (Bastin et al., 2017), while recordings from monkey PFC (Stoll et al., 2016) indicate that feedback-related activity in mPFC precedes activity in lateral PFC, suggesting a potential causal influence of ACC/mPFC activity on dlPFC activity. This possible causal chain of feedback processing (anterior insula →ACC/mPFC→dlPFC) echoes the cascade of uncertainty in the perception-action cycle (Fig. 1) and the regions observed in this study. Our results are broadly consistent with recent proposals that anterior insula serves as a “gatekeeper’ to the cognitive control network (Molnar-Szakacs and Uddin, 2022). By representing and updating state uncertainty estimates, anterior insula might contribute to downstream updates in outcome uncertainty estimates represented in dlPFC, with ACC/mPFC serving as a critical hub mediating this interaction (Alexander et al., 2017).
